# Chicago Journal of Nervous Diseases

**Published:** 1874-04

**Authors:** 


Chicago Journal of Nervous and Mental Disease.—We have received the two first numbers of this quarterly Journal of Nervous and Mental Diseases, and bid it a hearty welcome. Its Editors, Drs. Jewell & Bannister, seem bound to place before their readers the latest and best information obtainable, upon this subject. We wish them a full measure of success in their undertaking.

